# LncRNA AFAP1-AS1 exhibits oncogenic characteristics and promotes gemcitabine-resistance of cervical cancer cells through miR-7-5p/EGFR axis

**DOI:** 10.32604/or.2024.044547

**Published:** 2024-11-13

**Authors:** CHAOQUN WANG, TING ZHANG, CHAOHE ZHANG

**Affiliations:** 1 Department of Gynecology, Aviation General Hospital, Beijing, 100020, China; 2 Department of Burn and Skin Surgery, First Affiliated Hospital of Air Force Military Medical University, Xi’an, 710032, China; 3 Department of Oncology and Hematology, Second Hospital of Jilin University, Changchun, 130000, China

**Keywords:** Long non-coding RNA (lncRNA) AFAP1-AS1, miR-7-5p, Epidermal growth factor receptor (EGFR), Gemcitabine-resistance, Cervical cancer

## Abstract

**Background:**

Drug resistance is the main factor contributing to cancer recurrence and poor prognosis. Exploration of drug resistance-related mechanisms and effective therapeutic targets are the aim of molecular targeted therapy. In our study, the role of long non-coding RNA (lncRNA) AFAP1-AS1 in gemcitabine resistance and related mechanisms were explored in cervical cancer cells.

**Methods:**

Gemcitabine-resistant cervical cancer cell lines HT-3-Gem and SW756-Gem were constructed using the gemcitabine concentration gradient method. The overall survival rates and recurrence-free survival rates were evaluated by Kaplan-Meier analysis. The interaction was verified through a Dual-luciferase reporter gene assay and a Biotinylated RNA pull-down assay. Cell proliferation ability was assessed through methyl-thiazolyl-tetrazolium (MTT), soft agar, and colony formation experiments. Cell cycle and apoptosis were detected by flow cytometry.

**Results:**

Up-regulation of AFAP1-AS1 in cervical cancer predicted a poor prognosis. Besides, patients in the gemcitabine-resistance group had higher levels of AFAP1-AS1 than the gemcitabine-sensitive group. AFAP1-AS1 promoted tumor growth and induced gemcitabine tolerance of cervical cancer cells. In addition, AFAP1-AS1 mediated epidermal growth factor receptor (EGFR) expression by serving as a molecular sponge for microRNA-7a-5p (miR-7-5p). This present study also proved that the knockdown of EGFR or overexpression of miR-7a-5p abolished the accelerative role of AFAP1-AS1 overexpression in cancer progression and gemcitabine tolerance.

**Conclusions:**

In general, the AFAP1-AS1/miR-7-5p/EGFR axis was tightly related to the progression and gemcitabine tolerance of cervical cancer, providing potential targets for the management of cervical cancer.

## Introduction

Cervical cancer is the second most common malignancy after breast cancer in females, posing a major risk to the physical and mental well-being of women [1]. The occurrence of cervical cancer is closely linked to persistent high-risk human papillomavirus (HPV) infection [2]. Despite the use of cervical cancer vaccinations in clinical practice, not all high-risk HPV types are included in the current vaccines, and different locations have distinct HPV subtypes [3]. Consequently, thorough and efficient screening and treatment remain crucial. Radical hysterectomy, radiotherapy, chemotherapy, or a combination of them, are currently viable treatments for early-stage, locally progressed, and advanced cervical cancer. However, during the duration of treatment, approximately half of the patients continue to deteriorate. The main cause could be ascribed to the increase in the irreversible cell resistance of treatment [4,5]. Therefore, it is very important to probe the cervical cancer cell progression and drug resistance for better treatment of cervical cancer.

There is now more hope for the treatment of cervical cancer thanks to advancements in medical technology and the introduction of novel chemotherapeutic medications. Gemcitabine, one of the newest anticancer medications, stops deoxyribonucleic acid (DNA) synthesis by preventing the transition from the G1 phase to the S phase [6]. Gemcitabine has been widely used as a first-line treatment for non-small cell lung cancer and pancreatic cancer and as a second-line treatment for cervical cancer [7]. Research indicates that gemcitabine is an efficient inhibitor of both cell proliferation and tumor angiogenesis [8]. Unfortunately, there are currently no reliable methods for anticipating or tracking acquired gemcitabine resistance. At present, studies on gemcitabine resistance in cervical cancer are still very rare. Thus, it is vital to illuminate the molecular mechanism behind gemcitabine resistance to improve the effectiveness of cervical cancer treatment and guide the management of drug resistance in the future.

The function of long non-coding RNAs (lncRNAs) and microRNAs (miRNAs) on human diseases has steadily become clearer with the advancement of sequencing technology. Actin filament-associated protein 1-antisense RNA 1 (AFAP1-AS1) is closely associated with malignant tumors among many other cancer-related lncRNAs [9]. LncRNA AFAP1-AS1 with a length of 6810 bp located on chromosome 4p16.1 was first reported in esophageal adenocarcinoma in 2013 [10]. AFAP1-AS1, which is generated by the antisense direction of AFAP1 transcription is closely associated with the invasion and metastasis of tumor cells [11]. According to previous studies, AFAP1-AS1 is commonly upregulated in tumor tissues, and strongly correlative with the poor prognosis of cancer patients [12]. More importantly, earlier studies have explored the potential connection between AFAP1-AS1 and medication resistance. For instance, lncRNA AFAP1-AS1/miR-139-5p/RRM2 axis participates in non-small cell lung cancer cell proliferation and chemotherapy resistance [13]. However, the role and mechanisms of AFAP1-AS1 in chemotherapy resistance of cervical cancer have not been studied before.

The effects of AFAP1-AS1 on the *in vivo* and *in vitro* progression of cervical cancer were examined in this study. The role of AFAP1-AS1 on chemotherapy resistance to gemcitabine was also explored in cervical cancer cells. Besides, up-regulation of AFAP1-AS1 was closely related to the poor prognosis of cervical cancer patients and gemcitabine resistance of cervical cancer cells. Furthermore, epidermal growth factor receptor (EGFR) expression was relevant to the accelerative effects of AFAP1-AS1 in cervical cancer by serving as a molecular sponge for miR-7-5p. Taken together, AFAP1-AS1 exerts oncogenic characteristics and promotes gemcitabine resistance of cervical cancer cells via the miR-7-5p/EGFR axis.

## Materials and Methods

### Tissues collection

A pair of sixty samples of cervical cancer tissue and adjacent control tissues were collected from the patients who received surgery at Second Hospital of Jilin University between May 2016 and May 2019. The diagnosis and treatment of the patients were based on The Guidelines for the Diagnosis and Treatment of Common Gynecological Malignancies issued by the Gynecological Oncology Committee of China Anti-Cancer Association. Inclusion criteria: (1) Patients were diagnosed by the pathological examination. (2) Patients met the diagnostic criteria of cervical cancer [14]. Exclusion criteria: (1) Patients were accompanied with other cancers; (2) Patients without complete medical records. Tissue samples were further confirmed through the pathological examination and frozen in liquid nitrogen after surgical resection until use. According to the Response Evaluation Criteria in solid tumors [15], patients with stable disease or tumor regression (including complete remission and partial remission) after receiving gemcitabine-based on chemotherapy were considered as chemotherapy-sensitive group, while the rest patients were regarded as chemotherapy-resistant group. This study about patients was approved by the Medical Ethics Committee of Second Hospital of Jilin University (Approval number: 2016-JL-05) and executed in line with the Declaration of Helsinki. All patients signed informed consent (The informed consent was private).

### Prognosis and follow-up

**Figure 1:**
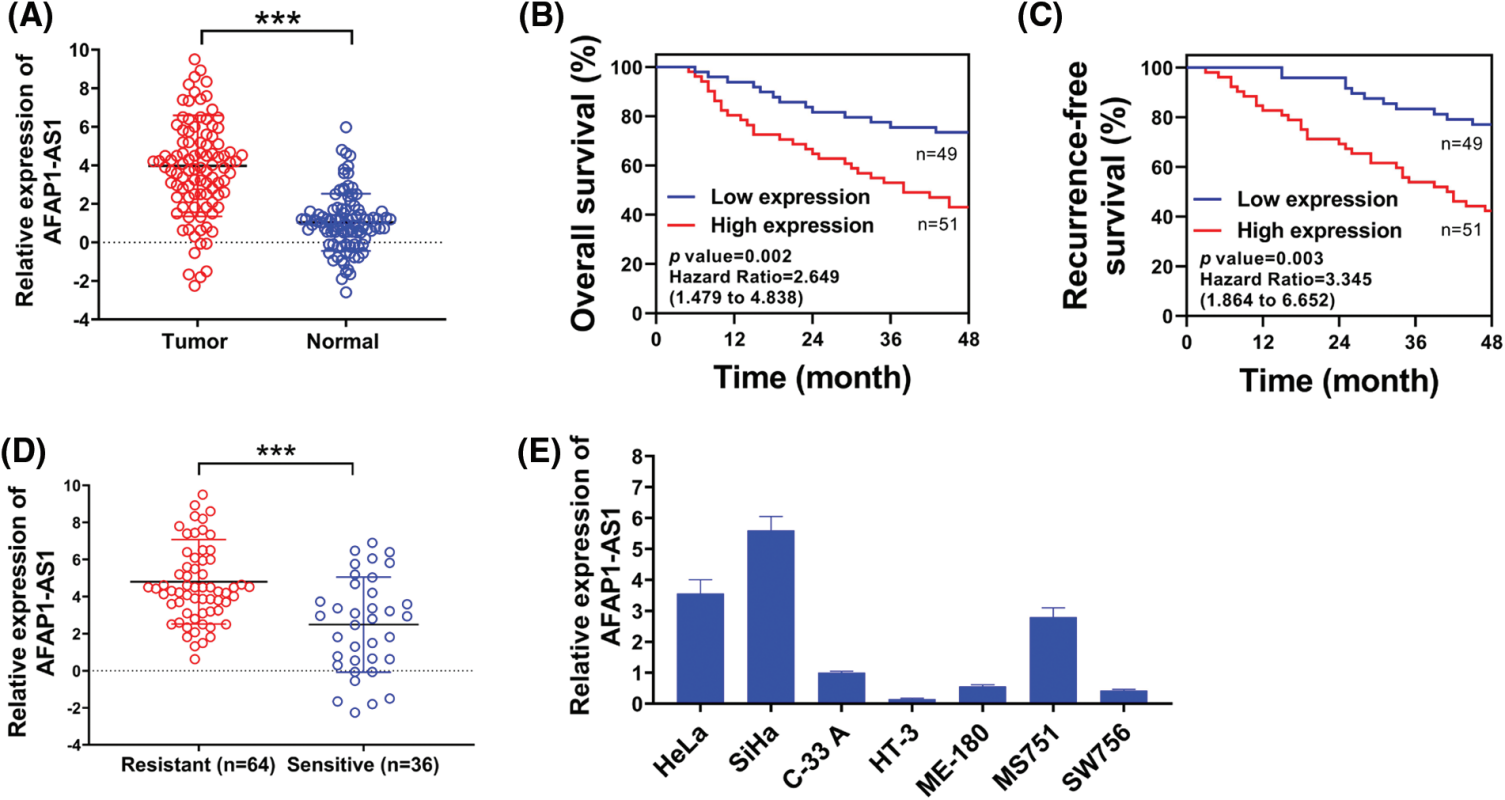
Up-regulated lncRNA AFAP1-AS1 was associated with chemo-resistance of cervical cancer cells. (A) Comparative expression of AFAP1-AS1 was assessed using qRT-PCR in 60 pairs of cervical cancer samples and control normal tissues. (B and C) Overall survival rates and recurrence-free survival rates of selected patients were shown via Kaplan-Meier survival analysis. (D) AFAP1-AS1 expression in the chemotherapy-resistance group (n = 43) or chemotherapy-sensitive (n = 17) group was examined through qRT-PCR. (E) qRT-PCR was used to detect the relative expression of AFAP1-AS1 in cervical cancer cell lines. ***p < 0.001.

The follow-up started from the discharge date. Total follow-up was conducted by an outpatient service through telephone interviews or medical record reviews. The survival time was counted in the unit of the month, and the end of observation of one patient was defined with one’s death. The overall survival rates and recurrence-free survival rates with the prolongation of the investigation time within 48 months were shown in the submitted Fig. 1.

### Kaplan-Meier analysis

The effects of 54,675 genes on survival in 21 cancer types were determined through the Kaplan-Meier plotter database [16]. Target genes were entered into the database. All selected cervical cancer cases were divided into the low-expression group and the high-expression group. Subsequently, Kaplan-Meier survival plots were generated. An automatically calculated hazard ratio with a 95% confidence interval and log rank *p*-value were obtained from the webpage. A difference was defined to be statistically significant when the log rank *p*-value was less than 0.05.

### Cell culture

Cervical cancer cell lines Hela (HPV positive), SiHa (HPV positive), C-33A (HPV negative), HT-3 (HPV negative), ME-180 (HPV positive), MS751 (HPV positive), and SW756 (HPV positive) were supplied from Chinese Academy of Sciences Cell Bank (Shanghai, China). The mycoplasma for cells was examined by using mycoplasma polymerase chain reaction (PCR) detection kit (Beyotime, Shanghai, China). The short tandem repeat (STR) profiling of cells was authenticated by the company before the cells bought from the company. The cell lines were grown in RPMI 1640 basic medium (Gibco, Rockville, MD, USA) containing 10% fetal bovine serum (FBS, Gibco, Rockville, MD, USA), 1% penicillin (Solarbio, Beijing, China), and 1% streptomycin (Solarbio, Beijing, China) at 37°C with 5% CO_2_.

### Transfection

AFAP1-AS1 expression vectors were constructed by Shanghai Jikai Gene Chemistry Technology Co., Ltd. (Shanghai, China) and verified by our laboratory staffs. An empty vector (EV) was used as a control. AFAP1-AS1 expression vectors (50 nM) and empty vectors (50 nM) were both transfected into cervical cancer cell lines using Lipofectamine 2000 (Invitrogen, Carlsbad, CA, USA). The primer sequences were as follows: sh-AFAP1-AS1-1 (sense: 5′-GUCCCAGCAUACACUCGUATT-3′, antisense: 5′-UACAAGUGUUAGCUGGAACTT-3′) sh-AFAP1-AS1-2 (sense: 5′-GGCCUUCAAUUUACAAGGATT-3′, antisense: 5′-UGCUUGUAAGUUGAAGCCATT-3′), miR-7-5p mimic (sense 5′-UGGUAGACUGGUGAUUUAGUUGUU-3′, antisense 5′-CAACAUAAUCACCAGUCCUACAUU-3′) and sh-EGFR (sense 5′-GGGCAAAUACAGCUUUGGACU-3′, antisense 5′-ACCAAAGCUGUAUUUGCCAGC-3′). Primers were designed and synthesized in Genscript (Nanjing, China). Transfection of miR-7-5p mimic (50 nM) or sh-EGFR (100 nM) was conducted through Lipofectamine 2000 (Invitrogen, Carlsbad, CA, USA).

### Quantitative real-time polymerase chain reaction (qRT-PCR)

The internal references for mRNA and miRNA were β-actin and U6 snRNA, respectively. Total ribonucleic acid (RNA) from cells was isolated using TRIzol (Invitrogen, Carlsbad, CA, USA). The PrimeScript RT Reagent Kit (TaKaRa, Dalian, China) was used to convert RNA (400 ng) into cDNA. The ABI PRISM 7900 Detection System (Applied Biosystems, Carlsbad, CA, USA) was used for the qRT-PCR assay. The qPCR reaction system included 2 × SYBR^®^ Green Realtime PCR Master Mix (Yeasen, Shanghai, China), 1 × primer mixture (final concentration: 0.4 mM), cDNA template (less than 10% of the total reaction system), and H_2_O (Yeasen, Shanghai, China). The cycle procedure was as follows: 95°C 2 min; 95°C 10 s, 55°C 15 s, 72°C 10 s, 40 cycles. Primer sequences were AFAP1-AS1 (forward: 5′-TCGCTCAATGGAGTGACGGCA-3′; reverse: 5′-GAATGCGTCTTCTTTCCCACG-3′), MiR-7-5p mimic (forward: 5′-GCGCTGGAAGACTAGTGATTTTGTTGTT-3′; reverse: 5′-AACGCTTCACGAATTTGCGT-3′), EGFR (forward: 5′-CTAAGATACCGTTCATC GCC-3′; reverse: 5′-GGACCGCACGACTTTGATCT-3′; The relative gene expression was quantified by the 2^−ΔΔCt^ method.

### Colony formation assay

HT-3 and SW756 cells were seeded in 6-well plates (1500 cells/well) and the culture media were replaced every three days. After three weeks, the visible colonies were fixed with 4% paraformaldehyde (Yeasen, Shanghai, China) for 15 min and stained with crystal violet (Yeasen, Shanghai, China) for 1.5 h at room temperature. Colonies were monitored and photographed using a fluorescence-inverted microscope (Olympus, Tokyo, Japan).

### Soft agar assay

The soft agar assay was conducted as the previous descriptions [17]. Briefly, HT-3 and SW756 cells in logarithmic growth phage were added into 3 mL RPMI (Gibco, Rockville, MD, USA) with 10% FBS (Gibco, Rockville, MD, USA) and 0.3% low-melting agarose (Bio-Rad, Hercules, CA, USA). Cells were grown for 3 weeks at 37°C with 5% CO_2_. Then, the colonies were stained, and counted using an FLx800 Fluorescence Microplate Reader (BioTek, Winooski, VT, USA).

### Construction of an in vivo model of cervical cancer

Animal experiments were approved by the Animal Care and Use Committees of Second Hospital of Jilin University (Approval number: 2022-YANSHEN-019) and conducted following the principles of the Guide for the Care and Use of Laboratory Animals. Six-week-old female BALB/c nude mice (15 ± 2) g were purchased from Vital River (Beijing, China), and fed with standard diet and water *ad libitum* at (25 ± 2)°C with 40%–60% the relative humidity and 12 h/12 h light-dark cycle. Mice were then randomly divided into empty vectors, AFAP1-AS1, AFAP1-AS1+MK-2206, sh-control, sh-AFAP1-AS1-1, and sh-AFAP1-AS1-2 groups with five mice in each group. Nude mice were subcutaneously injected with HT-3 cells (2 × 10^6^) transfected with empty vectors (EV), AFAP1-AS1 overexpression vectors, sh-control, sh-AFAP1-AS1-1, and sh-AFAP1-AS1-2. The tumor volumes were calculated every 3 days after injection according to the following equation: tumor volumes (mm^3^) = length × width^2^/2. Following 36 days, all the mice were sacrificed by the rapid cervical dislocation, tumor weight in each group was valued, and images of tumors were photographed. Besides, 120 mg/kg MK-2206 (MedChemExpress, Monmouth Junction, NJ, USA) was intraperitoneally injected into tumor-bearing mice with the overexpression of AFAP1-AS1-1, and monitored on days 2, 4, and 6 to resolve the role of AKT in the tumor cell growth.

### Construction of gemcitabine-resistance cervical cancer cells

HT-3 and SW756 cells were cultured in a culture medium containing gemcitabine (MedChemExpress, Monmouth Junction, NJ, USA) at escalating concentrations of 0, 1.25, 2.50, 5.00, 10.00, 20.00, 40.00, 80.00, 160.00 and 320.00 nM. In detail, HT-3 and SW756 cells were treated with 1.25 nM gemcitabine for 48 h, and then the medium was removed. Cells were restored in a new medium without gemcitabine for 72 h. Next, cells were treated with 2.50 nM gemcitabine for 48 h and restored for 72 h. The rest was done in the same manner until the treatment of 320.00 nM gemcitabine, to construct Gemcitabine-resistance cervical cancer cells HT-3-Gem and SW756-Gem. The drug resistance of HT-3-Gem and SW756-Gem cells was detected by methyl-thiazolyl-tetrazolium (MTT) assay.

### MTT assay

Trypsin was used to digest the cells, and the concentrations of cell suspension were adjusted to 5 × 10^4^/mL. Cells were then inoculated into the 96-well plates (100 μL/well) and cultured in a 5% CO_2_ incubator at 37°C. The next day, cells were transfected as indicated. 36 h after the transfection, 20 μL MTT reagent (5 mg/mL) (Yeasen, Shanghai, China) was mixed into each well and incubated for 4 h at 37°C. The supernatant was discarded, and 100 μL dimethyl sulfoxide (Yeasen, Shanghai, China) was appended into each well. Then, the plate was gently rocked to completely dissolve the crystal for ten minutes. Finally, the absorbance was measured at 405 nm using a Universal Microplate Spectrophotometer (BioTek, Winooski, VT, USA).

### Flow cytometry

Using the Annexin V-FITC apoptosis kit (Invitrogen, Carlsbad, CA, USA), HT-3 and SW756 cells were stained for 30 min at room temperature in the dark. Cell apoptosis was assessed using CellQuest software (BD Biosciences, San Jose, CA, USA) in keeping with standard procedures. Besides, the flow cytometry assay was used to examine the cell cycle following the immobilization of both cells with ice-cold 100% ethanol (Sigma-Aldrich, St. Louis, MO, USA) for one hour at 4°C, treatment by 100 μg/mL RNase A (Solarbio, Beijing, China) for one hour at 37°C, and a 30-min dark incubation period for 1000 μg/mL PI (Invitrogen, Carlsbad, CA, USA).

### Western blot

Total protein was collected after the cells were lysed with the lysis buffer (Solarbio, Beijing, China). The protein concentration was measured by BCA Protein Assay Kit (Enzyme-linked Biotechnology Co., Ltd., Shanghai, China). The protein samples (30 μg) were separated by using 10% sodium dodecyl sulfate-polyacrylamide gel electrophoresis (SDS-PAGE) equal, and then were electrophoretically printed using the semi-dry transfer printing method (100 V for 120 min) onto Polyvinylidene Fluoride membranes (Millipore, MA, USA) The membranes were blocked in 5% skim milk for one hour at room temperature. Primary antibodies against phosphorylated protein kinase B (pAKT) (1:1000, catalog number: sc-101629), AKT (1:500, catalog number: sc-377556), proliferating cell nuclear antigen (PCNA) (1:5000, catalog number: sc-25280), poly ADP-ribose polymerase (PARP) (1:500, catalog number: sc-365315), EGFR (1:200, catalog number: sc-373746) and glyceraldehyde-3-phosphate dehydrogenase (GAPDH) (1:5000, catalog number: sc-47724) (Santa Cruz Biotechnology, CA, USA) and cleaved caspase-3 (1:500, catalog number: ab2302), phosphorylated mitogen-activated protein kinase (pMAPK) (1:1000, catalog number: ab211061), MAPK (1:1000, catalog number: ab205926) (Abcam, Cambridge, UK) were then used to incubate with the membranes at 4°C overnight. The following day, a matching horseradish peroxidase-conjugated secondary antibody (Boster, Wuhan, China) was added to the membranes for incubation. Protein levels were normalized to GAPDH. The protein bands were visualized using enhanced chemiluminescence (Yeasen, Shanghai, China) and quantified by the ChemiDoc XRS imaging system (Bio-Rad, San Francisco, CA, USA).

### Bioinformatic prediction

The websites Miranda (http://mirtoolsgallery.tech/mirtoolsgallery/node/1055), TargetScan (http://www.targetscan.org/vert_71/), and the Microcosm Targets (https://tools4mirs.org/software/mirna_databases/microcosm-targets/) were used to predict the possible targets (accessed on 12 August 2022).

### Luciferase reporter assay

HT-3 cells were seeded in a 24-well plate, and then AFAP1-AS1 wild-type (WT) or AFAP1-AS1 mutant (MUT) combined with miR-7-5p mimic were co-transfected into HT-3 cells through Lipofectamine 2000 (Invitrogen, Carlsbad, CA, USA) to confirm the interaction between AFAP1-AS1 and miR-7-5p. Similarly, co-transfection of EGFR WT or EGFR MUT (100 ng) combined with miR-7-5p mimic (50 nM) was conducted on HT-3 cells to verify the interaction between EGFR and miR-7-5p. The Dual-Luciferase Reporter assay kit (Promega, Shanghai, China) was used to assess the luciferase activity after 24 h. The results were shown as the relative luciferase activity as calculated by firefly luciferase/Renilla luciferase.

### Biotinylated RNA pull-down assay

Following the instructions given previously [18], the pull-down experiment using biotinylated RNA was carried out. For miR-7-5p pulled down AFAP1-AS1, HT-3 cells with AFAP1-AS1 overexpression were transfected with biotinylated miR-7-5p WT, miR-7-5p MUT and negative control (NC) using Lipofectamine 2000. HT-3 cells expressing EGFR were transfected with biotinylated miR-7-5p WT, miR-7-5p MUT, and NC using Lipofectamine 2000 to downregulate EGFR using miR-7. Following 48 h, cell lysates were collected and incubated with Dynabeads M-280 Streptavidin (Invitrogen, Carlsbad, CA, USA) overnight at 4°C. Then, the beads were rinsed with ice-cold lysis buffer and high salt buffer. Finally, the bound AFAP1-AS1 and EGFR RNAs were extracted using TRIzol reagent (Invitrogen, Carlsbad, CA, USA) and the expressions were detected by qRT-PCR.

### Statistical analysis

At least three duplicates of each experiment were carried out. Student’s *t*-test was used to assess differences between means after data were analyzed using GraphPad Prism 8.0 software (GraphPad Inc., San Diego, CA, USA). The Student-Newman-Keuls post *hoc* was used to determine differences between various groups after the One-way ANOVA. A log-rank test was employed to evaluate the Kaplan-Meier analysis. A difference was defined to be statistically significant when it was *p* < 0.05, *p* < 0.01 or *p* < 0.001.

## Results

### Up-regulated lncRNA AFAP1-AS1 was correlated with poor prognosis and chemo-resistance in cervical cancer

AFAP1-AS1 expression in cervical cancer tissues (about 3-fold) is significantly higher than that in normal tissues (Fig. 1A). The relevance between the AFAP1-AS1 level and clinicopathological characteristics in patients with cervical cancer is exhibited in Table 1. Besides, patients with higher AFAP1-AS1 levels had lower overall survival rates (Fig. 1B) and recurrence-free survival rates (Fig. 1C) with the prolongation of the investigation time. In addition, patients in the chemotherapy-resistance group had much higher (about 4-fold) AFAP1-AS1 expression than patients in the chemotherapy-sensitive group (Fig. 1D). Besides, the AFAP1-AS1 expression in cervical cancer cell lines was evaluated in Fig. 1E. Thus, lncRNA AFAP1-AS1 was elevated and correlated with the poor prognosis and chemo-resistance in cervical cancer.

**Table 1:** Correlation between AFAP1-AS1 level and clinicopathological characteristics in cervical cancer patients

Characteristics	n	AFAP1-AS1 expression		*p* value
		Low	High	
Age (Years)				0.731
≥60	32	18	14	
<60	28	12	16	
Sex				0.556
Male	25	11	14	
Female	35	19	16	
Tumor size (mm)				0.256
≥30	40	17	23	
<30	20	13	7	
TNM stage				**0.001**
I--II	28	22	6	
III--IV	32	8	24	
Histological grade				0.238
Well	29	15	14	
Moderate-poor	31	15	16	
Lymph node metastasis				**0.008**
Positive	41	14	27	
Negative	19	16	3	
Distant metastasis				**0.012**
Positive	29	9	20	
Negative	31	21	10	

Note: The expression of AFAP1-AS1 was significantly related to distant metastasis, lymph node metastasis, and TNM stage (all *p* < 0.05), although it was not statistically correlated with histological grade, tumor size, sex, or age (all *p* > 0.05) in cervical cancer patients. Taken together, AFAP1-AS1 was highly expressed in cervical cancer patients with higher TNM stage, positive lymph node metastasis, and positive distant metastases. The bolded text in Table meant *p* < 0.05.

### LncRNA AFAP1-AS1 promoted the cervical cancer cell progression

**Figure 2:**
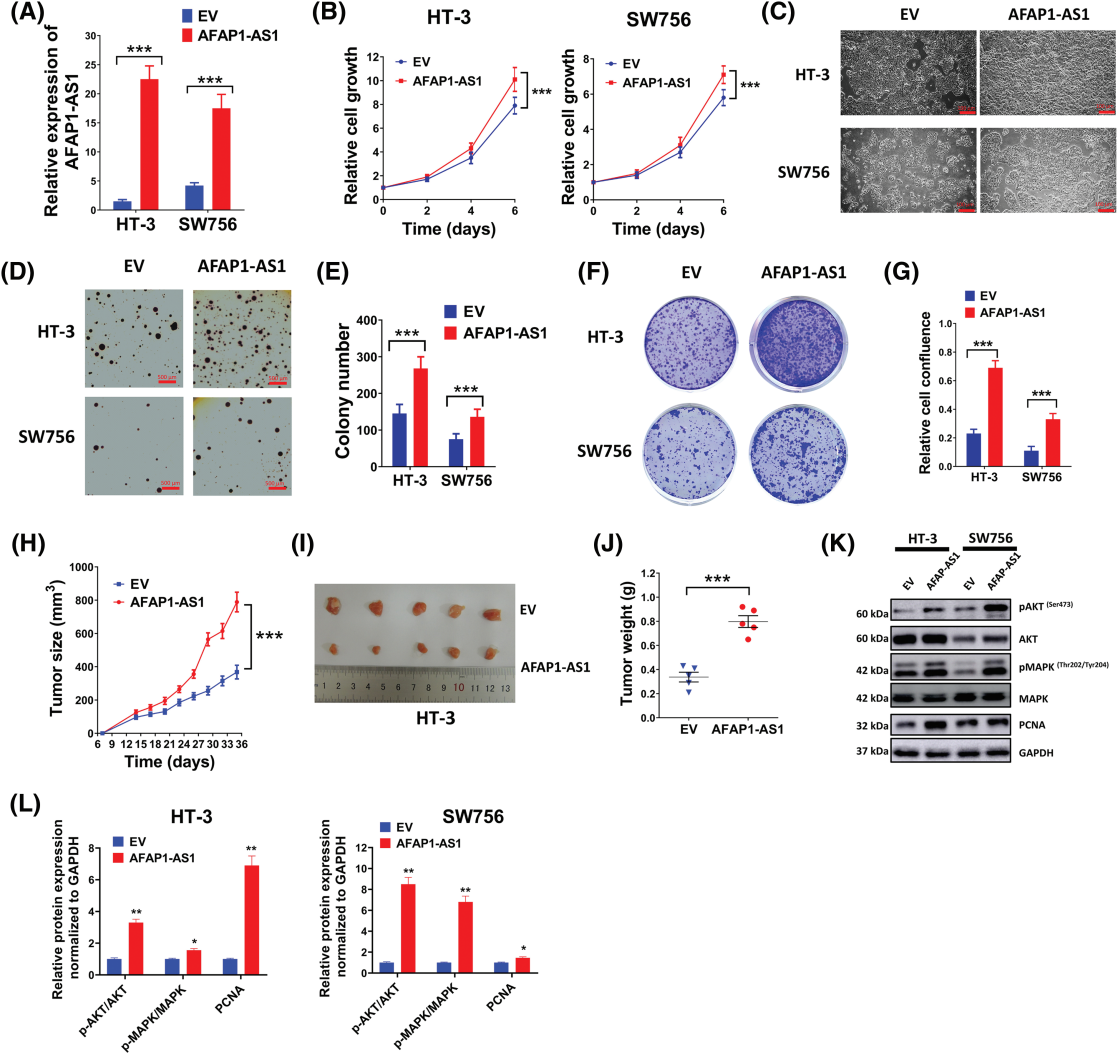
LncRNA AFAP1-AS1 boosted the progression of cervical cancer cells. Empty vectors (EV) and AFAP1-AS1 overexpression vectors were used to regulate the level of AFAP1-AS1. (A) The relative level of AFAP1-AS1 in HT-3 or SW756 cells was evaluated by qRT-PCR. (B) Cell growth was detected by MTT assay every two days in HT-3 or SW756 cells. (C) Representing images of cell growth conditions were shown. Scale bar = 100 µm. (D and E) Anchorage-independent growth ability was determined by soft agar assay. Representing pictures (D) and statistics of average colony number were shown (E). (F and G) Colony formation ability (F) of cells in different groups and relative cell confluence (G) were shown. (H–J) A nude mice model of cervical cancer was built by injecting with EV or AFAP1-AS1 expression vector pretreated HT-3 cells (2 × 10^6^). Tumor volume curve (H), representing tumor images (I) and tumor weight (J) were shown. (K and L) Expressions of PCNA, pMAPK, MAPK, pAKT, and AKT in HT-3 and SW756 cells were examined by western blot. Corresponding statistical results were also shown (L). *p < 0.05; **p < 0.01; ***p < 0.001.

To investigate the role of lncRNA AFAP1-AS1 in the development of cervical cancer cells, two cell lines HT-3 and SW756 were selected due to their relatively low expression of AFAP1-AS1 (Fig. 1E). The transfection of AFAP1-AS1 overexpression vectors increased the expression of AFAP1-AS1 in HT-3 and SW756 cells (Fig. 2A). Overexpressed AFAP1-AS1 markedly promoted cell growth (Fig. 2B,C). Moreover, overexpressed AFAP1-AS1 prominently elevated colony number and promoted cell confluence compared with the control (Fig. 2D–G). However, no statistical influence was discovered on the cell cycle between the control group and the overexpressed AFAP1-AS1 group (Fig. S1A). Also, overexpression of FAP1-AS1 had no statistical impact on cervical cancer cell stemness, as shown by no statistical difference in the level of NANOG and SOX2 between the control group and the overexpressed AFAP1-AS1 group (Fig. S1B). *In vivo* experiments further verified the proto-oncogene characteristics of AFAP1-AS1, as larger tumor size (Fig. 2H,I) and tumor weight (Fig. 2J) were found in AFAP1-AS1 overexpression mice compared to these in the control mice. Meanwhile, western blot results (Fig. 2K,L) showed that overexpressed AFAP1-AS1 prominently elevated the expression of PCNA and enhanced the phosphorylation of MAPK and AKT, indicating that AFAP1-AS1 promoted proliferation and the relationship between AFAP1-AS1 and MAPK signal pathway in cervical cancer. Moreover, the treatment of MK-2206, an inhibitor of AKT, markedly counteracted the promotion of HT-3 and SW756 cell growth caused by the upregulation of AFAP1-AS1 (Fig. S1C).

To further confirm the role of AFAP1-AS1 in cervical cancer, a loss-of-function study was conducted on SiHa lines with high expression of AFAP1-AS1. Silencing of AFAP1-AS1 markedly repressed cell growth (Fig. S2A). Moreover, the results of the colony formation assay (Fig. S2B,C) further showed that silencing of AFAP1-AS1 prominently reduced colony number and cell confluence relative to the control. The proto-oncogene characteristics of AFAP1-AS1 were also confirmed *in vivo*, as smaller tumor size (Fig. S2D,E) and tumor weight (Fig. S2F) were found in AFAP1-AS1 knockdown mice relative to these in the control mice.

### Higher expression of AFAP1-AS1 was related to gemcitabine resistance in cervical cancer cells

**Figure 3:**
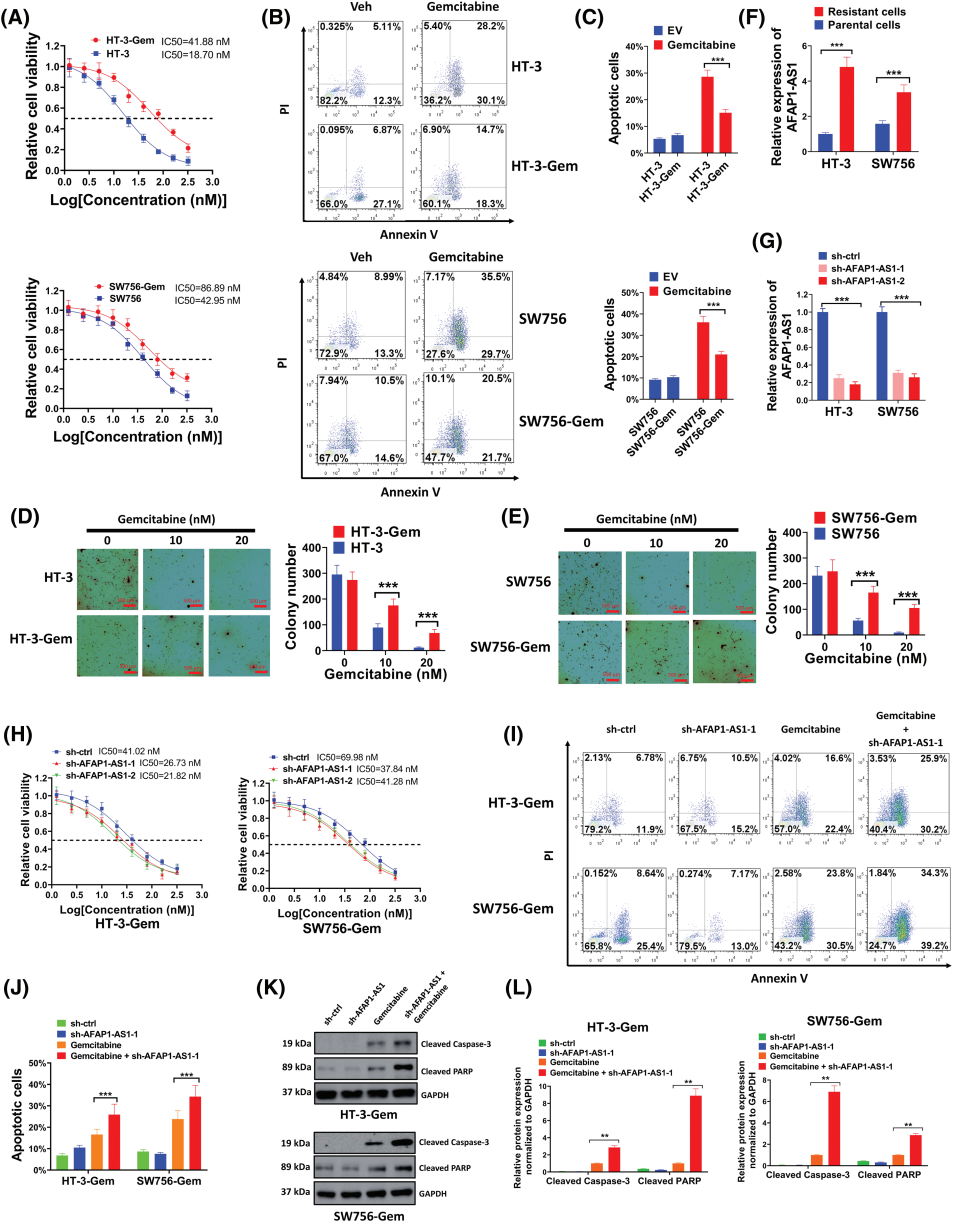
Higher expression of AFAP1-AS1 was interrelated with gemcitabine resistance in cervical cancer cells. (A) Gemcitabine-resistant cervical cancer cell lines HT-3-Gem and SW756-Gem were constructed and the cell viability was examined. (B and C) The detection of the effects of gemcitabine on HT-3/SW756 cells and HT-3-Gem/SW756-Gem cells by flow cytometry assay. (D and E) Soft agar assay (D) and statistics of average colony number (E) were conducted to compare anchorage-independent growth ability between HT-3-Gem and SW756-Gem and parent cells. (F) qRT-PCR was used to examine the expression of AFAP1-AS1 in resistant cells and parent cells. (G) The silencing effect of sh-AFAP1-AS1 was evaluated by qRT-PCR. (H) The function of sh-AFAP1-AS1 on gemcitabine resistance was assessed by MTT. (I and J) Flow cytometry was used to detect the cell apoptosis rate and was shown in the form of a bar chart. (K and L) Results of the western blot showed the expressions of cleaved-caspase-3 and cleaved-PARP. Corresponding statistical results were also shown (L). **p < 0.01; ***p < 0.001.

Gemcitabine-resistant cell lines, including HT-3-Gem and SW756-Gem were constructed, which had much higher IC50 than parental lines (Fig. 3A). Also, results of flow cytometry showed that gemcitabine had much better pro-apoptotic effect on HT-3 and SW756 cells than HT-3-Gem and SW756-Gem cells (Fig. 3B,C), further demonstrating that the drug-resistant cell lines were successfully constructed. Cell growth was significantly inhibited in HT-3 and SW756 cell lines compared with HT-3-Gem and SW756-Gem with increasing concentrations of gemcitabine (Fig. 3D,E). These above experiments confirmed that HT-3-Gem and SW756-Gem cell lines were successfully constructed. Moreover, a significantly elevated level of AFAP1-AS1 was discovered in resistant cells (Fig. 3F). For a more in-depth study, AFAP1-AS1 was silenced with sh-AFAP1-AS1-1 and sh-AFAP1-AS1-2 to downregulate the AFAP1-AS1 level (Fig. 3G). As expected, IC50 of sh-AFAP1-AS1 groups markedly declined compared with that of the sh-ctrl group (Fig. 3H). Since the two sh-AFAP1-AS1 had comparable inhibitory effects (Fig. 3G,H), sh-AFAP1-AS1-1 was chosen for the subsequent studies. In the following experiments, sh-AFAP1-AS1-1 further enhanced the pro-apoptotic effect of gemcitabine on HT-3-Gem and SW756-Gem cells (Fig. 3I,J). In addition, the expressions of cleaved-caspase-3 and cleaved-PARP were markedly elevated in the gemcitabine+sh-AFAP1-AS1-1 group compared with the other three groups (Fig. 3K,L). Thus, AFAP1-AS1 was closely associated with gemcitabine resistance in cervical cancer.

### Enforced AFAP1-AS1 expression increased gemcitabine tolerance in gemcitabine-sensitive cervical cancer cells

**Figure 4:**
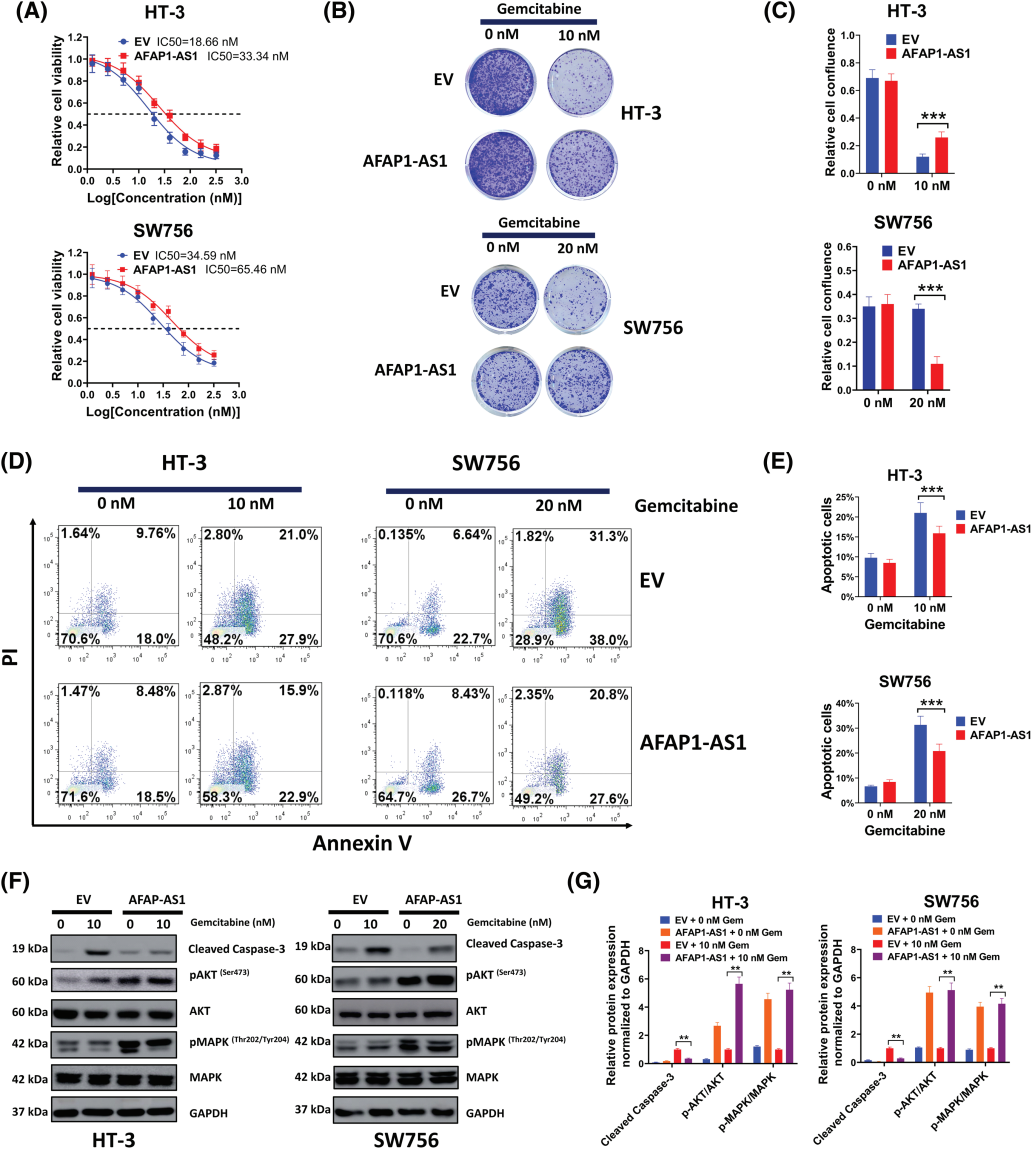
Enforced AFAP1-AS1 expression increased gemcitabine tolerance in gemcitabine-sensitive cervical cancer cells (A) Gemcitabine tolerance in each group was determined via MTT. (B) The function of enforced AFAP1-AS1 on cell growth was examined by colony formation assay. (C) The relative cell confluence of the AFAP1-AS1 group was analyzed. (D and E) The HT-3 cells were treated with 10 nM gemcitabine and SW756-Gem cells were incubated with 20 nM gemcitabine. Flow cytometry was conducted to assess the role of AFAP1-AS1 in gemcitabine for the induction of cell apoptosis. (F and G) Detection of expression of cell apoptosis and MAPK signal pathway-related proteins through western blot. Corresponding statistical results were also shown **p < 0.01; ***p < 0.001.

Based on the knowledge of the effect of AFAP1-AS1 downregulation on HT-3-Gem and SW756-Gem cells, comparable studies were carried out using gemcitabine-sensitive cervical cancer cells. As shown in Fig. 4A, the AFAP1-AS1 overexpression group had much higher IC50 compared to the control group. As expected, increasing concentration of gemcitabine had a weaker inhibitory effect on cell growth in the AFAP1-AS1 group (Fig. 4B) and the relative cell confluence in the AFAP1-AS1 group was also higher than that in the control (Fig. 4C). Then, the HT-3 cells were treated with 10 nM gemcitabine and SW756 cells were incubated with 20 nM gemcitabine, due to a higher IC50 in SW756 cells than that in HT-3 cells. Results of flow cytometry showed that the AFAP1-AS1 group had enhanced resistance to gemcitabine for the induction of cell apoptosis (Fig. 4D,E). In addition, overexpressed AFAP1-AS1 effectively weakened the function of gemcitabine on cell apoptosis and the activation of the MAPK signal pathway (Fig. 4F,G). Thus, overexpressed AFAP1-AS1 increased gemcitabine tolerance in gemcitabine-sensitive cervical cancer cells.

### LncRNA AFAP1-AS1 mediated the EGFR expression by serving as a molecular sponge for miR-7-5p

**Figure 5:**
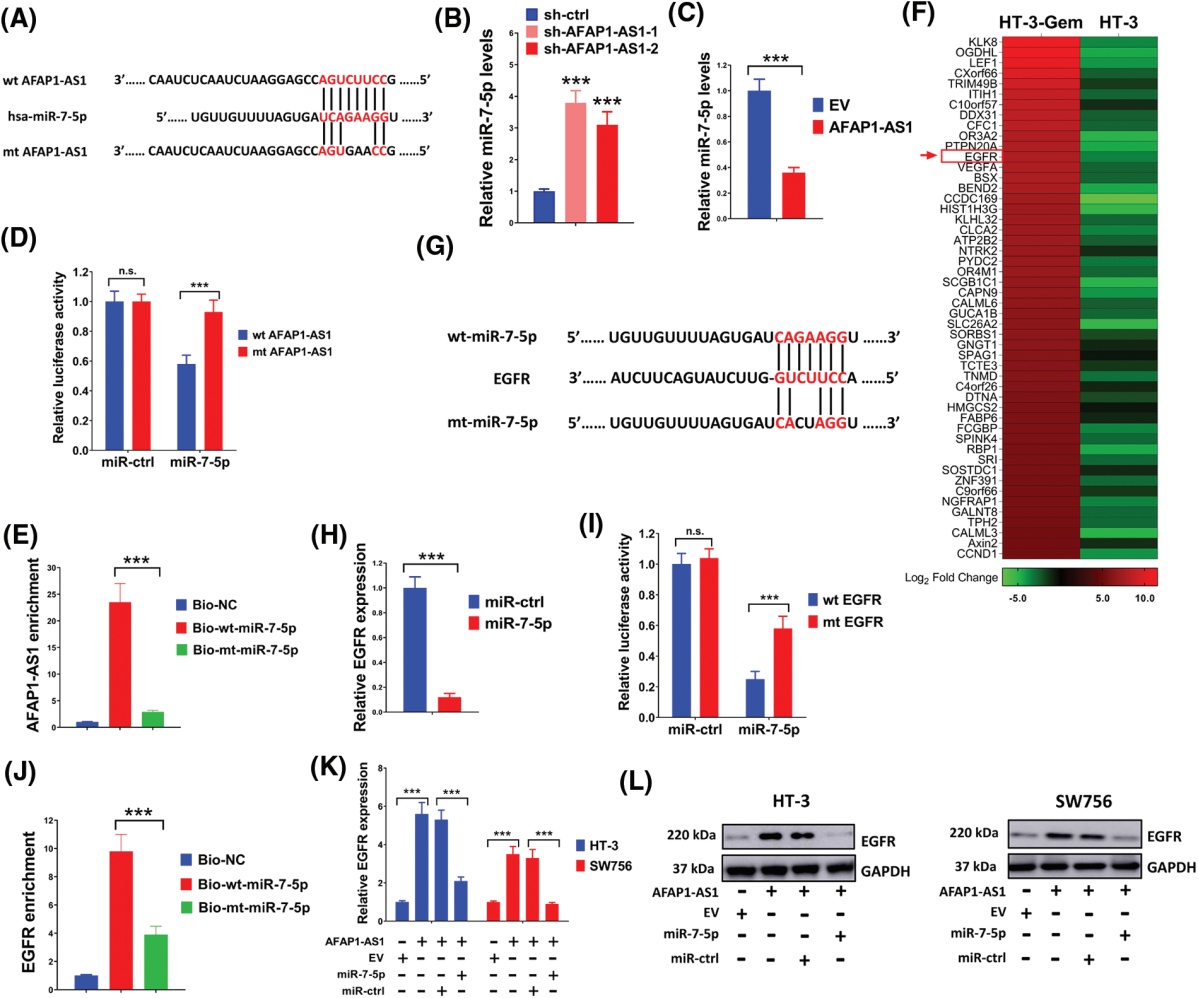
LncRNA AFAP1-AS1 mediated the EGFR expression by serving as a molecular sponge for miR-7-5p. (A) Bioinformatics analysis identified putative miR-7-5p binding sites with AFAP1-AS1. (B and C) Effects of AFAP1-AS1 on the expression of miR-7-5p were assessed through qRT-PCR. (D and E) To confirm the interaction between miR-7-5p and AFAP1-AS1, luciferase reporter assay and pull-down assay were conducted. (F) The heatmap was drawn to exhibit the expression of EGFR in HT-3-Gem cells and HT-3 cells. (G) Using bioinformatics prediction, potential binding sites between miR-7-5p and EGFR were identified. (H) Effects of miR-7-5p on the EGFR expression were evaluated through qRT-PCR. (I) The luciferase reporter examination evidenced the interaction between EGFR and miR-7-5p. (J) Pull-down assay showed an interaction between miR-7-5p and EGFR. (K and L) qRT-PCR and western blot were executed to verify the effects of AFAP1-AS1 and miR-7-5p on the expression of EGFR. n.s. *p >* 0.05; ***p < 0.001.

Possible binding sites between miR-7-5p and AFAP1-AS1 were predicted through bioinformatics analysis first (Fig. 5A). The level of miR-7-5p was significantly elevated by sh-AFAP1-AS1-1 and sh-AFAP1-AS1-2 and was strongly suppressed by AFAP1-AS1 overexpression vectors (Fig. 5B,C). The combination of miR-7-5p mimic and wide-type AFAP1-AS1 markedly reduced relative luciferase activity, but the alliance of miR-7-5p mimic and wild-type AFAP1-AS1 had little impact on luciferase activity, further indicating the direct interaction between miR-7-5p and AFAP1-AS1 (Fig. 5D). In addition, AFAP1-AS1 was successfully pulled down by biotinylated wild type miR-7-5p instead of mutant type miR-7-5p (Fig. 5E). The above results revealed the interaction between AFAP1-AS1 and miR-7-5p. Then we further explored the target gene of miR-7-5p. Abnormally high expression of EGFR in HT-3-Gem cells was detected as compared with that in the HT-3 cells (Fig. 5F). Similarly, possible binding sites between miR-7-5p and EGFR were forecasted first (Fig. 5G). In addition, the expression of EGFR was significantly repressed by miR-7-5p mimic (Fig. 5H) and the interaction between miR-7-5p and EGFR was further evidenced by luciferase reporter assay (Fig. 5I). Besides, EGFR was pulled down by biotinylated wild type miR-7-5p but not mutant type miR-7-5p (Fig. 5J). Moreover, the expression of EGFR could be elevated by overexpressed AFAP1-AS1, which was again suppressed by miR-7-5p mimic (Fig. 5K,L). Thus, the results of the above series of experiments showed that lncRNA AFAP1-AS1 mediated the EGFR expression by serving as a molecular sponge for miR-7-5p.

### The effects of the AFAP1-AS1/miR-7-5p/EGFR axis on cervical cancer cells

**Figure 6:**
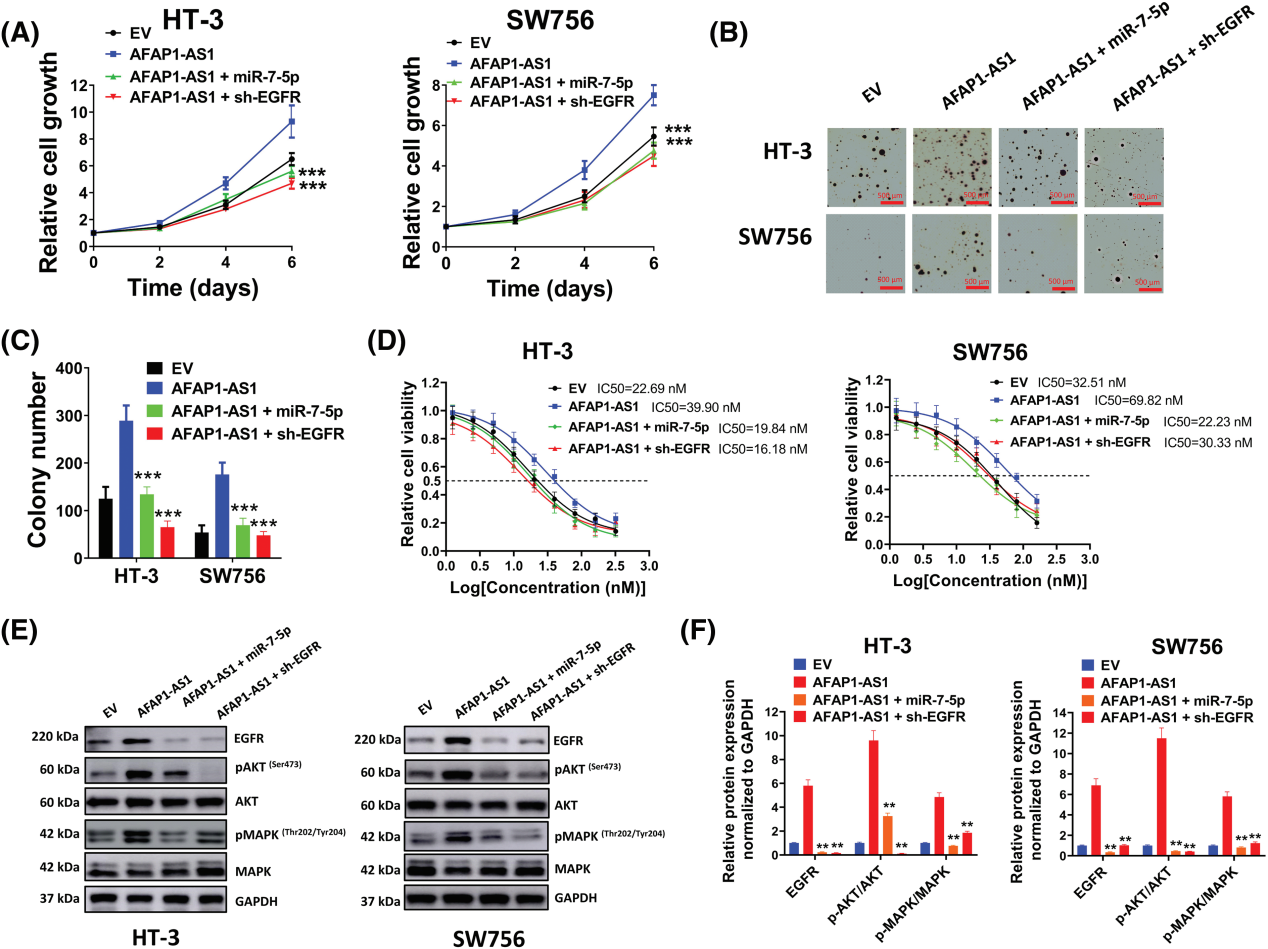
Effects of the AFAP1-AS1/miR-7-5p/EGFR axis on cervical cancer cells. (A) The cell growth rate was measured through an MTT assay. (B and C) Soft agar assay (B) and corresponding statistics of average colony number (C) showed effects of sh-EGFR or miR-7-5p mimic on tumor-promoting activity of AFAP1-AS1. (D) Drug resistance in each group was determined through MTT assay. (E and F) Expression of EGFR, pMAPK, MAPK, pAKT, and AKT was detected by western blot assay. Corresponding statistical results were also shown. **p < 0.01; ***p < 0.001.

To further verify interactions among AFAP1-AS1, miR-7-5p, and EGFR, the following experiments were conducted. Results in (Fig. 6A) showed that cell growth rate was prominently elevated by overexpressed AFAP1-AS1, which was markedly diminished by co-transfection of miR-7-5p mimic or sh-EGFR. Subsequent soft agar assay (Fig. 6B) and corresponding statistics of average colony number (Fig. 6C) also verified the suppressive effect of sh-EGFR or miR-7-5p mimic on the tumor-promoting activity of AFAP1-AS1. In addition, enhanced drug resistance by AFAP1-AS1 was availably weakened through miR-7-5p mimic or sh-EGFR (Fig. 6D). As expected, the expression of EGFR and phosphorylated AKT and MAPK were up-regulated by AFAP1-AS1, which were down-regulated by miR-7-5p mimic or sh-EGFR (Fig. 6E,F), further indicating the undertaking relationship among AFAP1-AS1, EGFR and miR-7-5p.

## Discussion

The occurrence and development of cervical cancer are related to many regulatory factors, including HPV infection, tumor formation, invasion, and drug resistance. Many genes implicated in tumor cell regulation, apoptosis, and drug resistance are currently the subject of extensive research. LncRNAs and miRNAs can not only be used as effective predictors of prognosis, recurrence, and metastasis but also as sensitive factors of chemotherapy and radiotherapy, whose regulation in the clinical efficacy could be largely improved [19]. Therefore, our study attempts to identify drug resistance-related genes in cervical cancer in the hopes of providing useful targets for cervical cancer treatment, given the paucity of drug resistance research in this field.

Previous research indicates that drug efflux of membrane transporters, autophagy, tumor microenvironment, epigenetics, tumor stem cells, and DNA damage repair processes are the key pathways associated with drug resistance [20–22]. In addition, noncoding RNA regulation (including miRNA and lncRNA) and drug resistance-related DNA methylation have also been extensively clarified in epigenetics [23,24]. An aberrant level of lncRNA AFAP1-AS1 is interrelated with a worse prognosis, an advanced clinical stage, and earlier tumor metastasis in various cancers [25]. Also, prior research has demonstrated that elevated AFAP1-AS1 is linked to a poor prognosis, as well as mobility and invasion of cervical cancer [26]. In our study, up-regulated AFAP1-AS1 in cervical cancer tissues was correlated with a poor prognosis. Additionally, gain-and loss-of-function results demonstrated that AFAP1-AS1 promoted cell growth *in vitro* and tumor xenograft formation *in vivo*. AFAP1-AS1/miR-320a/RBPJ axis is reported to be associated with stemness and chemoresistance to cisplatin in laryngeal carcinoma [27]. However, there are rather few studies about the function of AFAP1‑AS1 on drug resistance, especially in cervical cancer. Our work showed that the AFAP1-AS1 level was elevated in chemotherapy-resistant patients for the first time, which motivated us to look further for a relationship between AFAP1-AS1 and drug resistance in cervical cancer. The AFAP1-AS1 level was shown to be higher in resistant cells relative to sensitive cells after gemcitabine-resistant cervical cancer cells were created. Furthermore, upregulated AFAP1-AS1 expression enhanced the gemcitabine tolerance whereas suppressed AFAP1-AS1 attenuated the gemcitabine tolerance, which was comparable to the study on AFAP1-AS1 in esophageal squamous cell cancer [28]. Taken together, the upregulation of AFAP1-AS1 in cervical cancer was tightly interrelated with enhanced chemotherapy resistance.

Evidence has demonstrated that lncRNAs exert biological functions by acting as competing endogenous RNAs [29]. For example, lncRNA LINC00987 promotes adriamycin resistance in acute myeloid leukemia by sponging miR-4458 [30]. However, the same miRNA may serve as an oncogene or a tumor inhibitor in different human cancers or different stages of cancer progression. For example, miR-7-5p functions as a tumor inhibitor in many high incidences of malignant tumors, such as colorectal cancer [31] and non-small cell lung cancer [32]. However, Heverhagen et al. [33] report that miR-7-5p is up-regulated in the neuroendocrine neoplasms and acts as a potential biomarker for the poor prognosis. In the present study, miR-7-5p served as a target of AFAP1-AS1, and overexpressed AFAP1-AS1 effectively suppressed the miR-7-5p level. Also, the function of overexpressed AFAP1-AS1 on cell proliferation and gemcitabine tolerance was mitigated by the upregulation of miR-7-5p. Thus, our study revealed that overexpression of AFAP1-AS1 boosted cervical cancer progression and induced gemcitabine tolerance by suppressing miR-7-5p.

Previous research shows that the occurrence and development of cancers including cervical cancer are affected by abnormal expression of EGFR [34]. For example, Sun et al. [35] demonstrate that EGFR inhibitor effectively suppresses the human tongue cancer cell clone formation capacity and viability. Further studies reveal that mutations of receptor tyrosine kinases such as EGFR and c-Met lead to the over-activation of downstream signaling pathways, thus promoting tumor growth and metastasis [36]. Ras/Raf/MEK/ERK/MAPK axis and PI3K/AKT/mTOR pathway are the two main downstream signal transduction pathways of EGFR [37]. Autophagy is widely known to be strongly linked to drug resistance and cancer progression, and the study has also demonstrated that the EGFR-mediated RAS/RAF/MEK/ERK pathway regulates autophagy and is a critical factor in a variety of malignancies [38]. Therefore, it should be obvious that EGFR is important for the development of cancer. However, the association among lncRNA, miRNA, and EGFR in cervical cancer has not been widely probed. This study found that the interaction between miR-7-5p and EGFR, and overexpressed AFAP1-AS1 boosted the EGFR level by serving as a molecular sponge for miR-7-5p. In addition, the knockdown of EGFR attenuated the promoting effect of AFAP1-AS1 on cervical cancer development and gemcitabine tolerance. Moreover, results about the signaling pathways in this study showed that overexpressed AFAP1-AS1 induced the phosphorylation of AKT and MAPK. However, co-transfection of silenced EGFR or overexpressed miR-7a-5p both effectively blocked AFAP1-AS1-induced hyper-phosphorylation.

More importantly, overexpression or mutations of EGFR are frequently associated with increased drug resistance since EGFR is gradually becoming recognized as a biomarker of drug resistance in malignancies [39]. Due to rising treatment resistance, many current anti-EGFR-targeted agents are still insufficiently potent to stop the spread of tumors. Chang et al. [40] report that overexpression of TUSC7 suppresses chemotherapy resistance by sponging miR-224 via DESC1/EGFR/AKT signaling pathway in esophageal squamous cell carcinoma. In this study, AFAP1-AS1-induced gemcitabine tolerance was weakened in the presence of sh-EGFR. Thus, the future molecular targeted therapy should have a strong prohibitive function on the drug resistance of tumor cells.

However, there are still several limitations in this study. Firstly, although the results of the signaling pathways showed that AFAP1-AS1/miR-7-5p/EGFR axis induced the phosphorylation of AKT and MAPK in cervical cancer, the direct role of AKT and MAPK signaling pathway still needs to be confirmed by pharmacological block in the further study. Additionally, the role of AFAP1-AS1/miR-7-5p/EGFR axis in gemcitabine tolerance remains to be investigated in animal model of cervical cancer.

## Conclusion

In general, up-regulation of AFAP1-AS1 was interrelated with the progression and poor prognosis of cervical cancer patients. Besides, gemcitabine tolerance in cervical cancer cells was enhanced by the upregulation of AFAP1-AS1. Additionally, elevated expression of EGFR was promoted by overexpressed AFAP1-AS1, and the function of AFAP1-AS1 on cell development and drug resistance in cervical cancer cells were both reversed by the knockdown of EGFR or overexpression of miR-7-5p. Thus, the AFAP1-AS1/miR-7-5p/EGFR axis was closely associated with the progression and gemcitabine tolerance of cervical cancer cells, providing potential targets for the treatment of cervical cancer.

## Supplementary Materials

Figure. S1. Role of lncRNA AFAP1-AS1 in cervical cancer cell cycle and stemness. (A) Flow cytometry was used to identify the cell cycle after HT-3 and SW756 cells were overexpressed with AFAP1-AS1. (B) The relative protein level of NANOG and SOX2 was examined through western blot after HT-3 and SW756 cells were overexpressed with AFAP1-AS1. (C) The HT-3 and SW756 cell growth was monitored on days 2, 4, and 6 following the overexpression of AFAP1-AS1 as well as injection of MK-2206. ***p<0.001.

Figure. S2. LncRNA AFAP1-AS1 promoted the development of cervical cancer cells. (A) Cell growth was detected by the MTT experiment every two days in SiHa cells. (B and C) The colony formation ability of cells in different groups and the relative cell confluence was shown. (D-F) A nude mice model of cervical cancer was built by injecting with sh-control, AFAP1-AS1-1, and AFAP1-AS1-2 pretreated SiHa cells (2 × 106). Tumor volume curve (D), representing tumor images (E) and tumor weight (F) were shown. ***p<0.001.

## Data Availability

Upon a reasonable request, the corresponding author will provide the datasets used and/or analyzed in the current study.

## Supplementary Materials

The supplementary material is available online at https://doi.org/10.32604/or.2024.044547.
