# Supplementary figures and images for: LncRNA AFAP1-AS1 exhibits oncogenic characteristics and promotes gemcitabine-resistance of cervical cancer cells through miR-7-5p/EGFR axis
Source: Oncol Res. 2024 Nov 13;32(12):1867–79. doi: 10.32604/or.2024.044547 (PMC11576921; doi:10.32604/or.2024.044547)

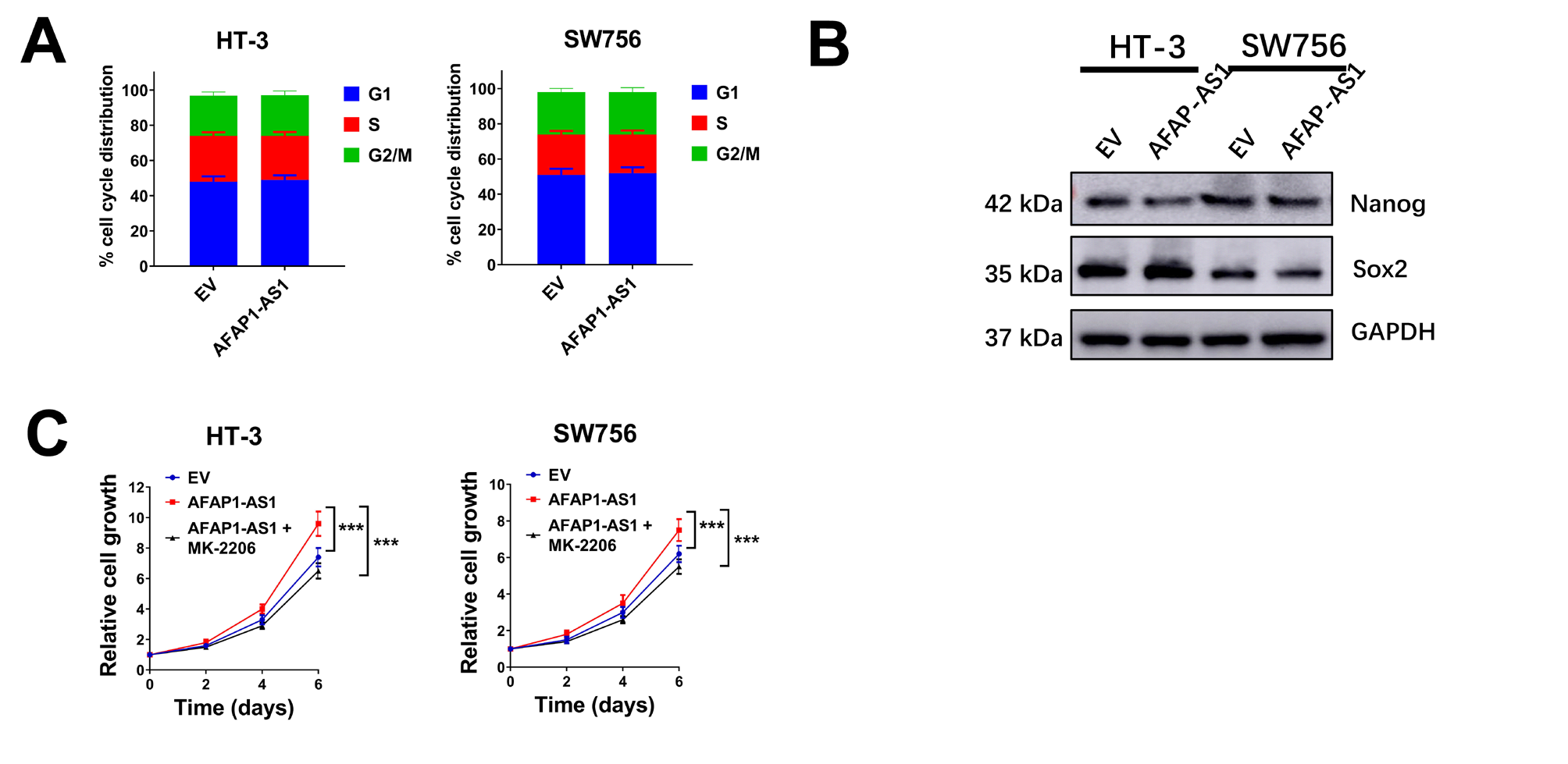

Supplement: Figure. S1. [file OncolRes-32-44547-s001.tif]

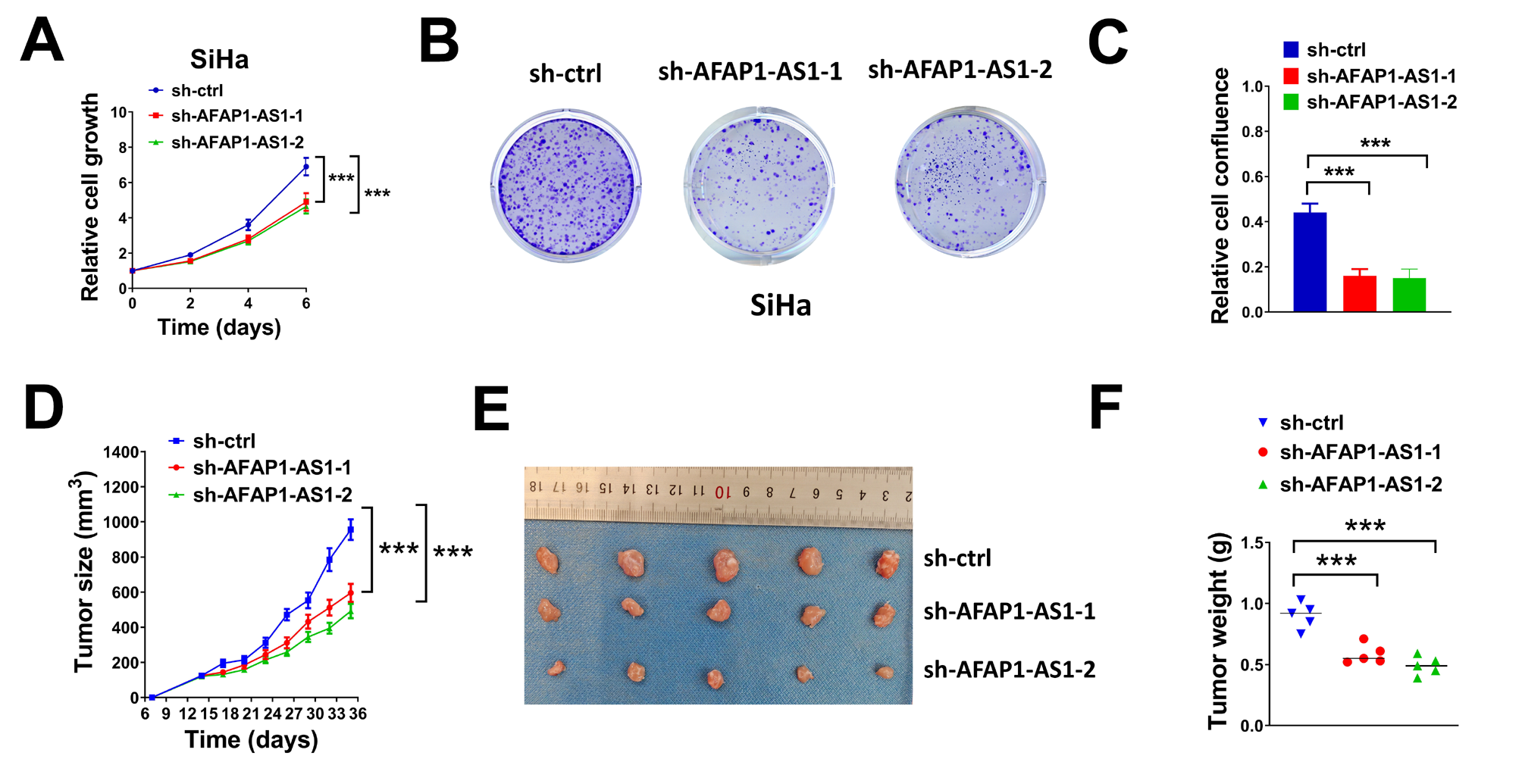

Supplement: Figure. S2. [file OncolRes-32-44547-s002.tif]
